# Cytoprotective effect of a functional antipollutant blend through reducing B[a] P-induced intracellular oxidative stress and UVA exposure

**DOI:** 10.3906/biy-1802-43

**Published:** 2018-10-25

**Authors:** Gizem ÖRS, Sultan GÜLÇE İZ

**Affiliations:** 1 Bioengineering Graduate Program, Institute of Natural and Applied Sciences, Ege University , Bornova, İzmir , Turkey; 2 Biomedical Technologies Graduate Program, Institute of Natural and Applied Sciences, Ege University , Bornova, İzmir , Turkey; 3 Department of Bioengineering, Faculty of Engineering, Ege University , Bornova, İzmir , Turkey

**Keywords:** Antipollutant, functional plant extracts, benzo[a]pyrene, reactive oxygen species, UVA, cytoprotective effect, cytotoxicity, cosmetics

## Abstract

Benzo[a]pyrene (B[a]P) is a ubiquitous environmental pollutant that reacts with skin and induces intracellular oxidative stress through reactive oxygen species (ROS) accumulation. The antipollution properties of natural extracts, especially including antioxidants, for inhibiting ROS in cells are gaining importance, in addition to the anticancer effects attributed to them. In this study, a commercial functional antipollutant blend of plant extracts consisting of ellagic acid standardized Punica granatum peel extract, Sambucus nigra fruit extract, Prunus cerasus seed extract, and hydrolyzed wheat protein with high antioxidant properties and UV damage-protective properties attributed to each one was investigated. The cytoprotective effect of this functional antipollutant blend was determined by ROS assay through reducing the level of intracellular ROS induced by B[a]P as an oxidative stress factor in human neonatal keratinocytes and fibroblast cells. In addition, the cytoprotective effect of the functional antipollutant blend after UVA exposure was also determined. It is shown that the oxidative damage induced by B[a]P and UVA, which are the most abundant factors of chemical and physical pollution, would be prevented by the functional antipollutant blend. Thus, it can be concluded that this antipollutant functional blend may offer a promising ingredient for the cosmetic industry's skincare products.

## 1. Introduction

Environmental factors such as temperature, climate, dryness (reduction of ambient temperature), heavy metals, air pollution (including mainly toxic gases), and exposure to sunlight (ultraviolet (UV) radiation) directly influence skin and hair health (Baudouin et al., 2002; López-Alarcón et al., 2013) . Most specifically, pollutants react with the skin, like polycyclic aromatic hydrocarbons (e.g., benzo[a] pyrene) and UV radiation that cause reactive oxygen species (ROS) accumulation (Narayanan et al., 2010) .

Environmental pollution, sunlight, and diet are the main causes of oxidative stress (Birch-Machin et al., 2010) . As environmental pollution is increasing significantly worldwide, the impact of pollutants on human health is a growing concern. People are exposed to motor vehicle emissions, fossil fuel combustion, forest fires, and industrial facilities extensively during their daily routines and B[a]P, which is known to be mutagenic and carcinogenic, is one of the main pollutants found in all smokes of incomplete combustion (Poon et al., 2012) . In addition, UV radiation emitted from the sun is one of the main causative agents of skin aging and cancer (Baudouin et al., 2002). As skin aging starts, cosmetic problems such as loss of elasticity and firmness of skin, wrinkles, age spots, inflammation, photoaging, hypersensitivity, hair loss, and dandruf occur (Naidoo et al., 2017) .

UV radiation is classified by energy and wavelength levels as UVA (320–400 nm), UVB (280–320 nm), and UVC (200–280 nm). Both UVA and UVB have been shown to contribute to skin aging; however, due to its ability to penetrate deeper into the dermis, UVA has been shown to cause more damage and is reported as the major reason for skin cancer (90%–99%) (Baudouin et al., 2002; Aslam et al., 2006; Birch-Machin et al., 2010; Baccarin et al., 2015) .

Consumption of natural antioxidants has been important for a healthy and long life for decades as they are considered as possible protection agents, since they reduce oxidative damage of the human body (Nemzer et al., 2014) . They interact with free radicals to transfer hydrogen to the interacting radical and reduce the reactivity of the hydrogen-transferring radical at the cellular level to prevent or delay oxidative stress. Anticancer effects attributed to natural plant antioxidants as safe therapeutics are now an emerging research field (Sevimli Gur et al., 2013) . In addition, it appears that the antioxidant formulations that eliminate the cosmetic problems induced by reactive oxygen species have created a new market as antipollution skincare products.

In this study, the antipollution properties of an antipollutant blend of natural extracts (PollufenceÔ) (Çalık Ekiz et al., 2016) , consisting of ellagic acid standardized Punica granatum peel extract, Sambucus nigra fruit extract, Prunus cerasus seed extract, and hydrolyzed wheat protein, was investigated. Punica granatum peel extract has high antioxidant activity and free radical-scavenging properties due to gallic acid and ellagic acid, respectively (Ismail et al., 2012) . Pomegranate peel increases type I procollagen synthesis, which was shown to help the regeneration of dermis in dermal fibroblasts (Aslam et al., 2006) . This reduces DNA damage and cell death caused by UVA and UVB (Baccarin et al., 2015) by reducing intracellular ROS formation (Viuda-Martos et al., 2010) . Sour cherry seeds (Prunus cerasus) are a source of tocochromanols and carotenoids (Daenen et al., 2008; Górnaś et al., 2016) with a high concentration of lipophilic compounds as antioxidants (Mittler et al., 2011) . Sambucus nigra contains polyphenolics with strong antioxidant activity that act as photoprotectives against UVA and UVB (Jarzycka et al., 2013) .

In this study, experimental sets for B[a]P and UVA exposure were established to investigate the activity of the functional antipollutant plant blend. As the pollutants are in contact mainly with skin and are used in sunscreens and shampoos, representative cell types of the skin were used in the experiments: noncancerous fibroblasts of the L929 cell line and keratinocytes of the HS2 cell line. L929 is commonly used in many experiments to evaluate material biocompatibility, drug cytotoxicity, and cell biology studies (Brétagnol et al., 2008) and it is the advised cell line in ISO-10993-5:2009 that describes test methods to assess the in vitro cytotoxicity of medical devices (ISO, 2009). It has been observed that the production of the ROS caused by B[a]P, which have carcinogenic and polluting effects, is reduced by the antipollutant blend. HS2 and L929 cells treated with antipollutant blend were found to be protected against UVA. Thus, the functional antipollutant blend is very promising to be used as an antioxidant and photoprotective agent for cosmetic industry.

## 2. Materials and methods

### 2.1. Chemicals and raw materials/test compounds

Used materials included DMEM/F12 (Sigma, D6421), FBS (Merck S0115), trypsin-EDTA (Sigma, T6689), L-glutamine (Biochrom, K0283), gentamycin (Merck A2712), PBS (Merck, L1825), DMSO (Sigma, D2650), MTT (Sigma M5655), and 96-well optical-bottom black plates (Thermo Scientific, 165305). B[a]P (CAS No. 50-328, MW 252.31 g/mol, ≥96% purity (HPLC)) was obtained from Sigma-Aldrich (B1760). B[a]P and the antipollutant blend were dissolved in DMSO and stock solutions were stored at –20 °C.

### 2.2. Maintenance of the cells

HS2 (human keratinocytes) and L929 (noncancerous mouse fibroblasts) were obtained from the Ege University Department of Bioengineering, Biotherapeutics and Biodiagnostics Laboratory, İzmir, Turkey. HS2 and L929 cells (passages between 10 and 12) were maintained in DMEM/F12 with 10% FBS, 2 mM L-glutamine, and 0.1% gentamycin in a humidified atmosphere with 5% CO 2 at 37 °C.

### 2.3. Determination of the effective concentration of the antipollutant blend

To determine the effective concentration (EC 50) of the antipollutant blend, prior to each treatment, 2 × 104 cells per well were seeded in a 96-well plate for 24 h and incubated to reach 90% confluence of the well surface. Cells were treated with different concentrations of antipollutant blend (0.75, 0.38, 0.19, 0.1, and 0.05 mg/mL) to determine the effective concentration. Each concentration was tested in triplicate; the final concentration of DMSO was ≤0.1% of the final volume so as not to cause any toxicity to cells. Both HS2 and L929 cells were maintained under the same experimental conditions. After 24 h of incubation, the MTT [3′-(4, 5-dimethylthiazol-2-yl)-2,5-diphenyl tetrazolium bromide] assay, which is a colorimetric cell viability assay, was performed. By measuring the reduction of MTT to formazan crystals the cell viability, which is an indicator of cytotoxic activity of test compounds, is determined. Briefly, the cell culture medium was discarded. MTT assay medium without serum, containing 0.5 mg/ mL MTT reagent, was added to each well and plates were incubated for 3 h at 37 °C and 5% CO . After that, 100 µL 2 of DMSO was added to each well to dissolve the formazan salts formed by the reduction of MTT by the metabolically active cells. The plates were read at 570 and 690 nm using a spectrophotometric microplate reader (Versamax Tunable Microplate Reader, VWR, USA). The EC 50 values of the antipollutant blend were calculated with GraphPad Prism v. 6.00 (GraphPad Software, USA).

### 2.4. Determination of inhibition concentration values of B[a]P and UVA

B[a]P was prepared as a concentrated 1000X stock solution and subsequently diluted with cell culture medium prior to use. Both HS2 and L929 cells were cultured at 37 °C for 24 h (Hockley et al., 2006) to find the concentration-dependent toxicity of B[a]P (20, 10, 5, 2.5, 1.25 µM) prepared at different concentrations (Hockley et al., 2006; Poon et al., 2012; Zhu et al., 2014) .

To find the time-dependent toxicity of UVA, cells were exposed to different doses of UVA at 366 nm at different time periods (30, 60, 180, 360 min) (Zhang et al., 2004) . To prevent any interaction with UVA, medium without phenol red was used. The distance between the UV source and the surface of the cultured cells was approximately 15 cm. All the experimental settings included control groups without any treatment. MTT assay was performed at the end of the incubation time. The inhibition concentration (IC50) of B[a]P and the lethal dose (LD50) of UVA were calculated with GraphPad Prism.

### 2.5. Cytoprotective effect of the antipollutant blend after B[a]P treatment measured by fluorescent intracellular oxidative stress

To determine the cytoprotective effect of the antipollutant blend, the cells were incubated with the antipollutant blend prior to B[a]P treatment or after B[a]P treatment to induce intracellular oxidative stress. After that, fluorescent measurementofintracellularoxidativestresswasperformed using the ROS assay kit (Oxiselect ROS assay kit, Cat. #STA 342, Cell BioLabs, USA) following the manufacturer’s instructions, which is a cell-based assay for measuring hydroxyl, peroxyl, or other reactive oxygen species activity within a cell. The assay employs the cell-permeable fluorogenic probe 2’,7’-dichlorodihydrouflorescein diacetate (DCFH-DA). Briefly, DCFH-DA is diffused into cells and is deacetylated by cellular esterases to nonfluorescent 2’,7’-dichlorodihydrouflorescein (DCFH), which is rapidly oxidized to highly fluorescent 2’,7’-dichlorodihydrouflorescein (DCF) by ROS. The fluorescence intensity is proportional to the ROS levels within the cell cytosol. The effects of antioxidant or free radical compounds on DCF-DA were measured against the control groups. The cells (2 × 10 4 per well) were seeded in 96-well optical-bottom black cell culture plates for 24 h in a CO2 incubator at 37 °C. The ROS levels in the cells treated with only antipollutant blend (24 h), B[a]P at the concentration of the IC50 (24 h) to cells incubated with antipollutant blend (24 h), or only B[a]P at a concentration of IC50 expected to induce ROS accumulation for 24 h were read kinetically at 485 nm excitation/518 nm emission per hour using a fluorescence plate reader (Fluoroskan Ascent, Thermo Scientific, Germany). Experiments were performed in triplicate. Microscopic images were taken under a fluorescence microscope (Zeiss HBO 50, Germany).

### 2.6. Determination of cytoprotective effects of the antipollutant blend following UVA exposure

The cytoprotective effects of the antipollutant blend after UVA exposure for both HS2 and L929 cells were determined with different experimental settings of the antipollution blend treatment prior to UV exposure or after UV exposure. Cells incubated with 0.1% DMSO and cells without any treatment were used as control groups. DMSO was used as a solvent for the antipollution blend. After 24 h of treatment, the MTT assay was performed as described previously in Section 2.3.

### 2.7. Statistical analyses

All experiments were carried out at least in triplicate and data were expressed as mean ± standard deviation (SD). The variables between treated and control samples were compared using two-way ANOVA. In all the cases, P < 0.05 was considered significant.

## 3. Results and discussion

### 3.1. Dose-dependent cellular toxicity

**Figure 1:**
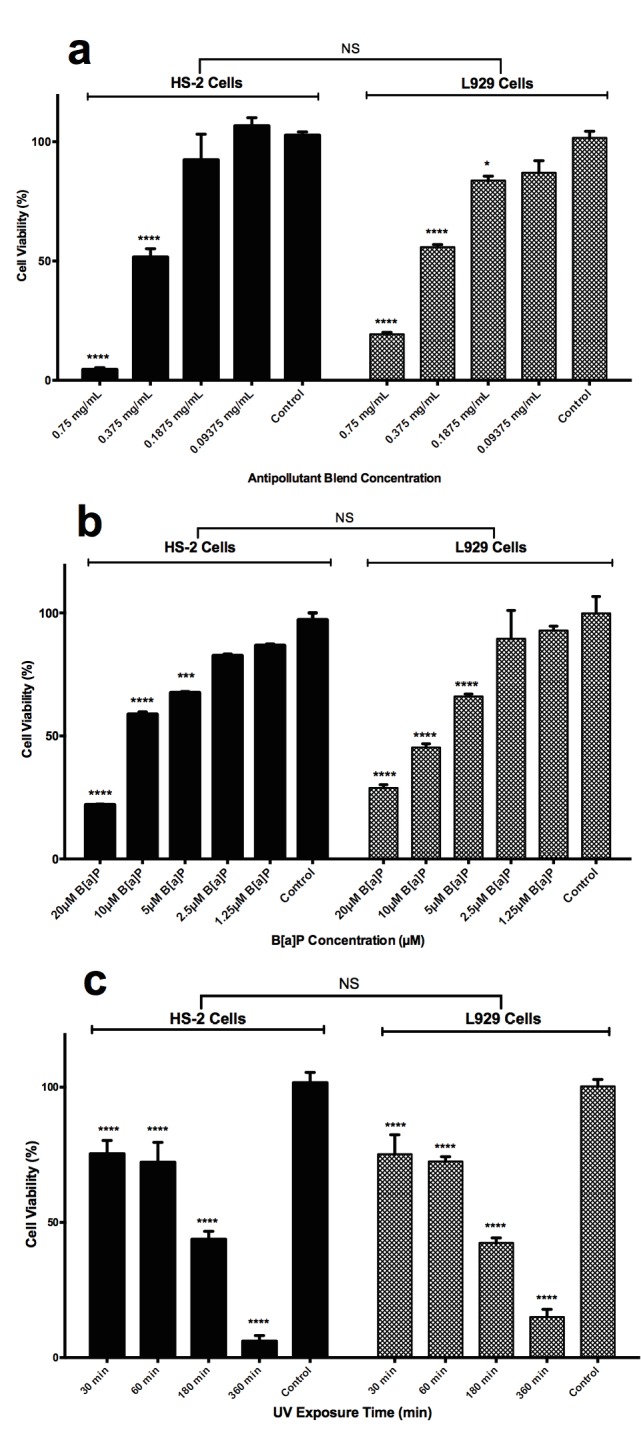
Determination of effective concentration (EC50) values after antipollutant blend treatment (a), inhibition concentration (IC50) value for B[a]P (b), and lethal dose (LD50) value for UVA (c) exposure for HS2 and L929 cells as determined by MTT cell viability assay. Statistical significances are presented as treatment groups compared to nontreated cell controls without any treatments. a) HS2 and L929 cell viability measured by MTT assay after treatment with different concentrations of antipollutant blend for 24 h. b) HS2 and L929 cell viability measured by MTT assay treated with different concentrations of B[a]P for 24 h. c) HS2 and L929 cell viability measured by MTT assay after different doses of UVA. Data are presented as mean ± SD. Statistical significance: nonsignificant (NS), P > 0.05; * P < 0.05; ** P < 0.01; *** P < 0.001; **** P < 0.0001.

To determine any potential antipollutant activity of the plant extract, the nontoxic effective concentration of the extract must be identified. For this reason, the concentrationdependent cellular toxicity was measured by MTT assay. A wide range of antipollutant blend concentrations were assayed (0.75 mg/mL to 0.09375 mg/mL), as shown in Figure 1a. Due to the rich antioxidant features of the functional antipollution plant blend tested in this study, grape seed, a known antioxidant, was used as a positive control (Katiyar, 2008). eThre was not a significant difference between the cell lines in the means of cytotoxicity (P > 0.05). However, both HS2 and L929 cells at 0.75 mg/mL and 0.375 mg/ mL concentrations showed significant decreases in cell viability compared to controls (P < 0.001). As the cell viability was inversely correlated with the concentration of the antipollutant plant extract, 0.09375 mg/mL (≈0.1 mg/mL) concentration, which provides over 90% cell viability both for HS2 and L929, was chosen to be used in other experimental sets to determine the cytoprotective activity after B[a]P or UVA treatment. B[a]P is one of the most toxic polycyclic aromatic hydrocarbons. It produces active compounds that form DNA adducts and induces ROS production, leading to the occurrence of oxidative DNA damage (Hecht et al., 1993). This compound is metabolized by cytochrome P450 (CYP450) enzymes. After being converted to B[a]P-7,8-diol-9,10-epoxide (BPDE) by cytochrome P450, it is covalently linked to DNA to cause mutagenic and carcinogenic effects. Besides DNA adducts, the induction of oxidative stress is another molecular mechanism of B[a]P-induced cytotoxicity. Excessive ROS showed carcinogenic, teratogenic, and mutagenic potential in cells. The cytotoxicity of B[a]P was measured by the MTT assay at 24 h after treatment. As shown in Figure 1b, B[a]P reduced the viability of both cell lines in a concentration-dependent manner (P < 0.05); however, there was no significant difference between the viability of the two cell lines (P > 0.05). The percentages of viable cells observed after exposure to B[a]P (20, 10, 5, 2.5, 1.25 µM) were 22.52 ± 1.644%, 56.15 ± 0.8975%, 72.96 ± 1.017%, 81.36 ± 1.201%, and 85.57 ± 1.312% for HS2 cells and 23.10 ± 5.604%, 56.52 ± 3.059%, 73.22 ± 3.467%, 81.58 ± 4.095%, and 85.75 ± 4.472% for L929 cells after 24 h. In a concentration-dependent manner, the cell viabilities increased when the concentration of B[a]P decreased. B[a]P at concentrations of >10 µM induced significant cell death in cells compared with controls. Therefore, the concentration of B[a]P of 10 µM was selected for the ROS induction.

It was shown that B[a]P has a concentration- and timedependent cytotoxicity (Zhang et al., 2004; Hockley et al., 2006; Poon et al., 2012; Zhu et al. 2014) . Hockley et al. (2006) investigated the cytotoxicity of B[a]P in time- (6, 24, 48 h) and dose-dependent (0.01, 0.10, 0.25, 0.50, 1.00, 2.50, or 5.00 µM) manners in MCF7 and HEPG2 cells. No cytotoxic activity was detected for 6 h of treatment of this substance while high cytotoxicity was found for 24 h or 48 h of treatment. However, it was mentioned that at 24 h and 48 h of treatment the concentration does not make a significant difference (Hockley et al., 2006). Zhu et al. (2014) investigated cytotoxicity in time- (24, 48 h) and concentration-dependent (0–80 µM) manners in lung epithelial cells (BEAS-2B) and reported that B[a] P has concentration-dependent cytotoxicity. B[a]P at concentrations greater than 5 µM induced significant cytotoxicity in BEAS-2B cells compared to the control. Treatment of cells with 5 µM B[a]P induced a 20% increase in ROS production compared to the control (P < 0.05) (Zhu et al., 2014) . Poon et al. (2012) used human dermal fibroblast (HDF) cells to be administered 0, 0.1, 1, 10, 30, and 50 µM B[a]P concentrations. It was determined that DNA damage and cell death were significant in HDF cells followed by 10 µM B[a]P treatment, which also resulted in approximately 75% cell viability for the cytotoxicity and genotoxicity assays (Poon et al., 2012) .

Zhang et al. (2004) investigated the production of ROS exposed to B[a]P and UVA radiation in KB cells (subline of the ubiquitous keratin-forming tumor cell line HeLa). UVA was applied to the cells for a maximum of 120 min. They determined that effective ROS production was about 25 min (Zhang et al., 2004) . To find the time-dependent toxicity of UVA, the cells were exposed to different doses of UVA at 366 nm for different time periods (30, 60, 180, 360 min). The cytotoxic effects of UVA exposure time on HS2 and L929 cells were assessed using the MTT assay. As shown in Figure 1c, UVA reduced the viability of the two cell lines in a time-dependent manner (P < 0.005); however, there was not a significant difference between the cell lines. The percentages of viable cells observed after UVA irradiation (30, 60, 180, 360 min) were 76.77 ± 1.903%, 70.32 ± 1.686%, 44.54 ± 1.486%, and 5.870 ± 2.954% on HS2 cells and 74.96 ± 2.217%, 69.29 ± 1.967%, 46.58 ± 1.661%, and 12.52 ± 3.216% on L929 cells as shown in Figure 1c. The LD 50 values for UVA exposure were calculated as 111 min and 115.3 min for HS2 and L929 cells, respectively. UVA exposure time of 60 min induced 30% significant cell death (P < 0.0001) for both cells compared to controls, as shown in Figure 1c. For this reason, the UVA exposure time for the following experimental design was chosen to be 60 min.

## 3.2. Determination of antipollution effect of the natural plant extract after B[a]P and UVA exposure

B[a]P is an environmental pollutant used to induce intracellular oxidative stress (Zhang et al., 2004) and genotoxicity (Gao et al., 2005) for in vitro experimental models. It was also proven that using B[a]P and UVA simultaneously increases the intracellular ROS (Gao et al., 2005).

**Figure 2:**
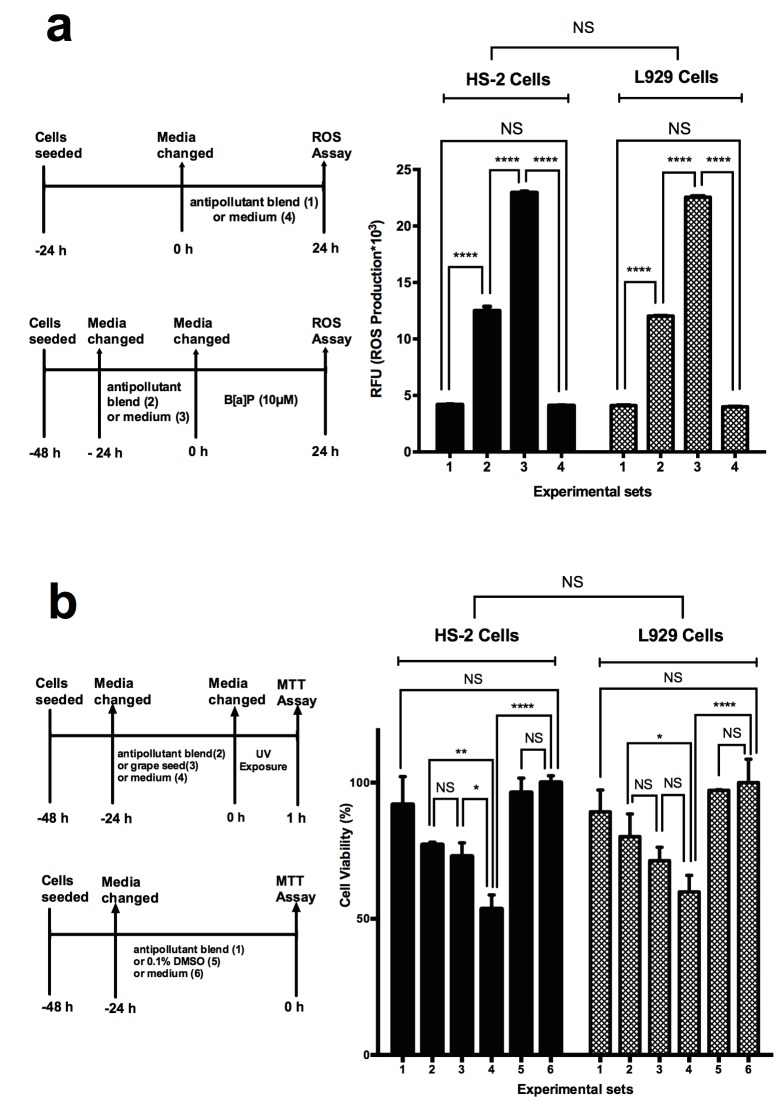
Determination of antipollution effect of the natural plant extract on HS2 cells and L929 cells after B[a]P and UVA exposure by ROS assay and MTT assay. a) ROS assay results: 1st group, antipollutant blend treatment only for 24 h; 2nd group, 24 h of B[a]P exposure to cells treated with antipollutant blend prior to B[a]P for 24 h; 3rd group, cells incubated with B[a]P for 24 h as control of group 2; 4th group, cells incubated with only medium as control of group 1. Data are presented as mean ± SD. Statistical significance: **** P < 0.0001. b) MTT assay results: 1st group, cells incubated with 24 h of antipollution blend only; 2nd group, 1 h of UV exposure to cells prior to MTT assay, which were incubated with antipollution blend for 24 h; 3rd group, 1 h of UV exposure to cells prior to MTT assay, which were incubated with grape seeds for 24 h; 4th group, 1 h of UV exposure to cells prior to MTT assay, which were incubated with medium for 24 h; 5th group, cells incubated with 0.1% DMSO control for 24 h as control of group 1, 6th group, cells incubated with medium only for 24 h as control. Data are presented as mean ± SD. Statistical significance: nonsignificant (NS), P > 0.05; * P < 0.05; ** P < 0.01; *** P < 0.001; **** P < 0.0001.

In this study, the oxidation of DCFH-DA to DCF was used to measure ROS levels. According to the results of the ROS assay (Figure 2a), the production of ROS in antipollutant blend-treated cells (Group 1) was not significantly different than in the cells without any treatment (Group 4). Cells treated with antipollutant blend for 24 h prior to 24 h of B[a]P treatment (Group 2) had a significantly lower amount of ROS (approximately 13,000 RFU) compared to control cells with a ROS accumulation of 23,000 RFU without any antipollutant blend treatment (Group 3) (P < 0.0001) (Figure 2a).

In particular, B[a]P and UVA are pollutants that increase oxidative stress by the free radicals they form to lead to skin aging and ultimately skin cancer. Skin cancer accounts for about 40% of all newly diagnosed cancers each year (Bowden, 2004). As the skin is exposed to many environmental carcinogens like polycyclic aromatic hydrocarbons (like B[a]P), various metals, and UV as a first barrier, UV has been studied extensively regarding the formation of skin cancer (de Gruijl et al., 1993). UV radiation is a physical pollutant responsible for the majority of skin cancers in the human body (Baudouin et al., 2002). The genotoxicity of UV light has been noted in many studies (Zhang et al., 2004) and both UVA and UVB have been reported to induce ROS production in keratinocytes (Baccarin et al., 2015). However, UVA is primarily associated with increased levels of ROS (Zhang et al., 2004; Bossi et al., 2008) . ROS are produced by endogenous photosensitizers of UV, blocking of antioxidant activity, and inflammation of the dermis (Gao et al., 2005; Reeg et al., 2015) . As shown in Figure 2b, the UVA protection efficacy of the antipollutant blend was determined. For this purpose, UVA was applied to the cells that were treated with antipollutant blend (Group 2). As a positive control, grape seed extract, which has UVA protection properties (Katiyar, 2008) (Group 3), and blank control cells with only UVA treatment (Group 4) were used. In addition, groups without any UVA exposure with antipollutant blend treatment (Group 1), with 0.01% DMSO in cell culture medium (Group 5), and without any treatment (Group 6) were used (Figure 2b). The viabilities of cells treated with the antipollutant blend prior to UVA treatment were around 80% (Group 2), as in the group of positive control cells treated with grape seed, which had viability of 75% (Group 3) with a nonsignificant difference. However, cells without any treatment had viability of 50% in Group 4 with a significant difference compared to Group 2 (P < 0.01).

**Figure 3:**
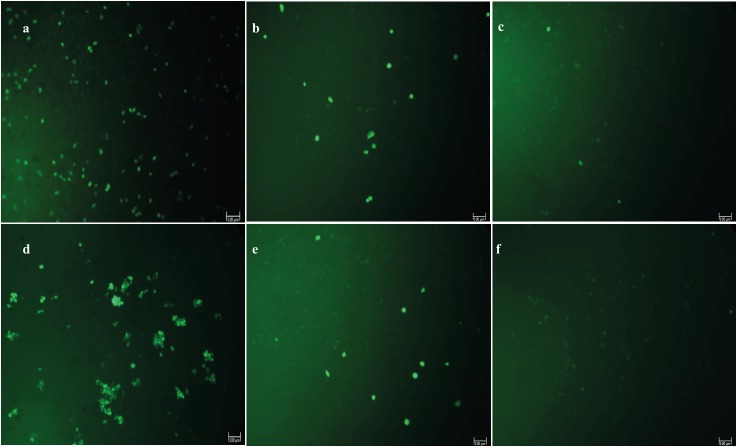
Microscopic images for DCF fluorescent staining proportional to ROS accumulation. a) HS2 cells treated with B[a]P for 24 h, b) HS2 cells incubated with antipollutant blend for 24 h prior to 24 h of B[a]P exposure, c) untreated HS2 cells, d) L929 cells treated with B[a]P for 24 h, e) L929 cells incubated with antipollutant blend for 24 h prior to 24 h of B[a]P exposure, f: untreated L929 cells.

The microscopic images for DCF fluorescent staining proportional to ROS accumulation are seen in Figure 3. Figure 3a corresponds to the experimental setting of Group 2 in Figure 2a, Figure 3b to Group 3, and Figure 3c to Group 4 of HS2 cells. In the same manner, Figure 3d, Figure 3e, and Figure 3f correspond to Groups 2, 3, and 4 of L929 in Figure 2a. As seen in Figure 3a, HS2 cells treated with B[a]P for 24 h, compared to HS2 cells incubated with the antipollutant blend for 24 h prior to 24 h of B[a] P exposure, have a significantly higher (P < 0.0001) ROS accumulation, as also shown in Figure 2a when Group 2 and Group 3 are compared. The same pattern is seen with L929 cells for Figure 3d and Figure 3e. In other words, in a qualitative manner, the microscopic images for DCF fluorescent staining proportional to ROS accumulation given in Figure 3 support the fluorimetric quantitation in Figure 2a. Overall, the treatment of HS2 and L929 cells with B[a]P at 10 µM for 24 h significantly increased cellular ROS levels, as indicated by green fluorescence as seen in Figure 3a and Figure 3d for HS2 and L929 cells, respectively.

This study presents research strategies developed to investigate the mechanisms and potential roles of the antipollutant blend against chemical and physical pollutants. Previous research showed that polycyclic aromatic hydrocarbon B[a]P (Zhang et al., 2004; Poon et al., 2012) increased the formation of 8-hydroxy-2’-deoxyguanosine in cultured cells. It is assumed that the underlying mechanism is the production of ROS by photosensitization, and ROS are represented by a family of oxygen-based derivatives including hydrogen peroxide and hydroxyl radicals (Mittler et al., 2011). This study demonstrates that B[a]P significantly potentiates the generation of ROS in neonatal human keratinocytes and fibroblast cells, as seen in Figure 2a and Figure 3. In this study, ROS is detected with the DCF reagent because in biological systems the probes must react with antioxidants and generate signals to be quantified very quickly (Myhre et al., 2003). DCFH-DA is the most commonly used probe for detection of intracellular H2O2 and oxidative stress (Gomes et al., 2005). The probes are acetylated to easily penetrate the cell membrane; cytoplasmic esterases cleave the acetyl group and then liberate DCF, which can react with intracellular reactive species (Myhre et al., 2003; Pavelescu, 2015) . It can be diffused into the cell membrane and is naturally hydrolyzed with endogenous cell esterases, which yield diacetate groups and form DCFH. They are a good display for hydroxyl radicals (HO), hydrogen peroxide (H2O ), and per2 oxyl radicals (ROO) (Hempel et al., 1999). Oxidative stress and redox signaling pathways are known to be induced by ROS production, which may lead to apoptosis/necrosis, aging, and/or disease (Schieber et al., 2014) .

**Figure 4:**
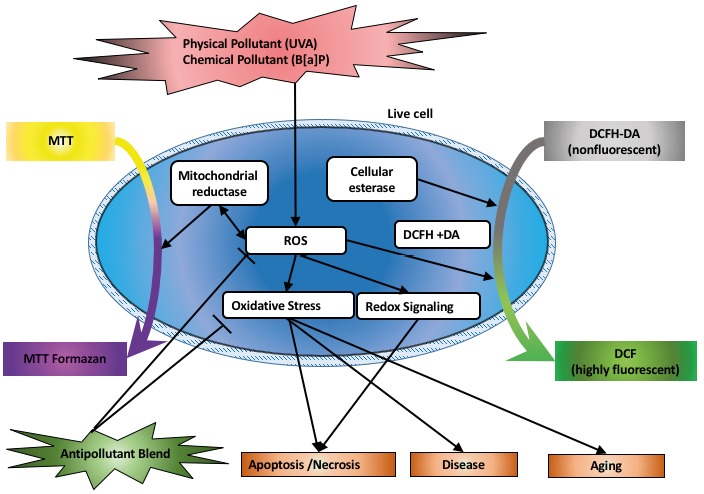
The induction of reactive oxygen species generation in this study after B[a]P and UVA exposure as chemical and physical pollutants, respectively, and the assays used to measure possible outcomes of the antipollutant blend treatment.

In this study, the antipollution properties of an antipollutant blend of natural extracts, PollufenceÔ (Çalık Ekiz et al., 2016), consisting of ellagic acid standardized Punica granatum peel extract, Sambucus nigra fruit extract, Prunus cerasus seed extract, and hydrolyzed wheat protein, were investigated. Antioxidant and skin cancer treatment potentials of the functional natural blend are the interests of this study. One of the ingredients of the antipollutant natural extract in this study is ellagic acid standardized pomegranate peel, which has been used for several treatment and prevention purposes for inflammation, diabetes, diarrhea, dysentery, dental plaque, and intestinal infections and malarial parasites, as reviewed extensively by Ismail et al. (2012). The antioxidant activity of pomegranate peel is associated with its wide range of phenolic compounds. Several studies have confirmed the cytoprotective effects of ellagic acid from pomegranate peel for oxidatively injured living cells, and an increase in the ellagic acid content increases its antioxidant activity (Seeram et al., 2004) . In addition, several studies showed that ellagic acid decreases the ROS generation in human fibroblast cells after UVA and UVB exposure (Pacheco-Palencia et al., 2008) . The other ingredient, sour cherry seeds (Prunus cerasus), is a rich source of phenolic antioxidants, especially anthocyanins (Toydemir et al., 2013) . The third ingredient of the natural antipollutant blend used in this study is Sambucus nigra extract, which contains polyphenolics, which are very promising natural compound candidates for sunscreens because they can absorb a broad spectrum of UV radiation including the UVA and UVB regions. Moreover, they reduce the penetration of the radiation into the skin and decrease inflammation, oxidative stress, and DNA-damaging effects (Jarzycka et al., 2013). In this study, the possible protective effects of the antipollutant blend treatment against oxidative damage induced by B[a]P and UVA exposure were determined by MTT and DCFH-DA assays through the mitochondrial reductase and cellular esterase of living cells, respectively, as summarized in Figure 4. The major findings of our study are as follows: both B[a]P and UVA exposure inhibits cell proliferation; B[a]P induces ROS, thus increasing oxidative stress both in HS2 and L929 cells; the antipollutant blend decreases the accumulation of ROS caused by B[A] P treatment in vitro both for HS2 and L929 cells; and antipollutant blend treatment prior to UVA exposure protects cells against UVA exposure both in HS2 and L929 cells. The findings of this study support that the cytoprotective effects of the functional antipollutant blend by reducing B[a]P induced intracellular oxidative stress and UVA exposure.

In our highly industrialized and chemically polluted world, natural products are gaining importance for extensive health benetfis from antiinflammatory and anticancer effects to antiinfective and photoprotective effects. The antipollutant blend used in our study consisting of Sambucus nigra, Punica granatum, and Prunus cerasus extracts is a very promising functional blend because of the antioxidant and photoprotective effects attributed to each ingredient along with our findings. Considering our results, it can be suggested that this antipollutant functional blend may be a good ingredient for skincare products for the cosmetic industry due to their antioxidant and UV protection properties, which may especially alter skin aging.

## Acknowledgment

The antipollutant blend (Pollufence TM) used in this study was provided by Bionorm Natural Products Co., Ltd.
